# Author Correction: The importance of Antarctic krill in biogeochemical cycles

**DOI:** 10.1038/s41467-019-13390-0

**Published:** 2019-11-20

**Authors:** E. L. Cavan, A. Belcher, A. Atkinson, S. L. Hill, S. Kawaguchi, S. McCormack, B. Meyer, S. Nicol, L. Ratnarajah, K. Schmidt, D. K. Steinberg, G. A. Tarling, P. W. Boyd

**Affiliations:** 1 Institute for Marine and Antarctic Studies, University of Tasmania, Hobart, TAS Australia; 2 British Antarctic Survey, Natural Environment Research Council, High Cross, Madingley Rd, Cambridge, CB3 0ET UK; 3 Plymouth Marine Laboratory, Prospect Place, The Hoe, Plymouth, PL1 3DH UK; 4 Australian Antarctic Division, Kingston, TAS Australia; 5 Antarctic Climate and Ecosystems CRC, University of Tasmania, Hobart, TAS Australia; 6 Alfred Wegener Institute for Polar and Marine Research, Bremerhaven, Germany; 7 Institute for Chemistry and Biology of the Marine Environment, University of Oldenburg, Carl-von-Ossietzky-Straße 9-11, 26111 Oldenburg, Germany; 8 Helmholtz Institute for Functional Marine Biodiversity at the University of Oldenburg, Ammerländer Heerstrasse 231, 26129 Oldenburg, Germany; 9 Department of Earth, Ocean and Ecological Sciences, University of Liverpool, Liverpool, UK; 10 School of Geography, Earth and Environmental Science, University of Plymouth, Plymouth, UK; 11 Virginia Institute of Marine Science, College of William & Mary, Williamsburg, VA USA; 12 Present Address: Department of Life Sciences, Imperial College London, Silwood Park Campus, Buckhurst Road, Ascot, Berkshire, SL5 7PY UK

**Keywords:** Carbon cycle, Ecosystem services, Environmental impact, Marine biology

Correction to: *Nature Communications* 10.1038/s41467-019-12668-7, published online 18 October 2019.

The original version of this Article included an incorrect competing interests statement, which read ‘The authors declare no competing interests’, rather than the correct ‘The authors declare the following competing interest: Steve Nicol has been employed to provide scientific advice to the Association of Responsible Krill harvesting companies.’ This has been corrected in both the PDF and HTML versions of the Article.

